# Pneumococcal Septic Arthritis among Adults, France, 2010–2018

**DOI:** 10.3201/eid3101.240321

**Published:** 2025-01

**Authors:** Farida Hamdad, Nadim El Bayeh, Gabriel Auger, Olivia Peuchant, Frédéric Wallet, Raymond Ruimy, Florence Reibel, Christian Martin, Marie-Cécile Ploy, Frédéric Robin, Chrislène Laurens, Philippe Lanotte, Marie Kempf, Jennifer Tetu, Hélène Revillet, Isabelle Patry, Philippe Cailloux, Mélissa Azouaou, Emmanuelle Varon, Pierre Duhaut, Alain Lozniewski, Vincent Cattoir

**Affiliations:** Université de Picardie Jules Verne, Amiens, France (F. Hamdad, N. El Bayeh, P. Duhaut); Centre Hospitalier Universitaire de Brabois-Nancy, Nancy, France (F. Hamdad, P. Cailloux, A. Lozniewski); Centre Hospitalier Universitaire Amiens-Picardie, Amiens (N. El Bayeh, P. Duhaut); Centre Hospitalier Universitaire CHU de Rennes, Rennes, France (G. Auger, V. Cattoir); Centre Hospitalier Universitaire de Bordeaux, Bordeaux, France (O. Peuchant); Centre Hospitalier Universitaire de Lille, Lille, France (F. Wallet); Centre Hospitalier Universitaire de Nice, Nice, France (R. Ruimy); Centre Hospitalier Universitaire Henri Mondor, Créteil, France (F. Reibel); Centre Hospitalier Universitaire de Limoges, Limoges, France (C. Martin, M.C. Ploy); Centre Hospitalier Universitaire de Clermont Ferrand, Clermont Ferrand, France (F. Robin); Centre Hospitalier Universitaire de Montpellier, Montpellier, France (C. Laurens); Centre Hospitalier Universitaire de Tours, Tours, France (P. Lanotte); Centre Hospitalier Universitaire d’Angers, Angers, France (M. Kempf); Centre Hospitalier Universitaire de Dijon, Dijon, France (J. Tetu); Centre Hospitalier Universitaire de Toulouse, Toulouse, France (H. Revillet); Centre Hospitalier Universitaire de Besançon, Besançon, France (I. Patry); Centre Hospitalier Intercommunal de Créteil, Créteil, France (M. Azouaou, E. Varon)

**Keywords:** *Streptococcus pneumoniae*, joint infection, septic arthritis, adult, bacteria, France, antimicrobial resistance, vaccines

## Abstract

*Streptococcus pneumoniae* infection is considered an uncommon cause of arthritis in adults. To determine the clinical and microbiological characteristics of pneumococcal septic arthritis, we retrospectively studied a large series of cases among adult patients during the 2010–2018 conjugate vaccine era in France. We identified 110 patients (56 women, 54 men; mean age 65 years), and cases included 82 native joint infections and 28 prosthetic joint infections. Most commonly affected were the knee (50/110) and hip (25/110). Concomitant pneumococcal infections were found in 37.2% (38/102) and bacteremia in 57.3% (55/96) of patients, and underlying conditions were noted for 81.4% (83/102). Mortality rate was 9.4% (8/85). The proportion of strains not susceptible to penicillin was 29.1% (32/110). Of the 55 serotyped strains, 31 (56.4%) were covered by standard pneumococcal vaccines; however, several nonvaccine serotypes (mainly 23B, 24F, and 15A) had emerged, for which susceptibility to β-lactams was low.

Septic arthritis is a serious infectious disease caused by invasion of microorganisms (most commonly bacteria) into the synovial membranes and resulting in purulent joint effusion. It constitutes a medical emergency and is associated with high morbidity and mortality rates (*1*–*4*). In industrialized countries, the annual incidence of proven or probable septic arthritis is ≈4–10 cases/100,000 general population but among persons with rheumatoid arthritis or other underlying joint disease is significantly higher (30–70 cases/100,000 population) (*1*–*4*). The increased prevalence of septic arthritis over recent decades might be associated with population aging, wider use of immunosuppressive drugs, and the growing number of invasive orthopedic and prosthetic procedures (*1*–*4*).

The pathogen most frequently involved in septic arthritis is *Staphylococcus aureus*, followed by *Streptococcus* spp. (mainly β-hemolytic streptococci and, more rarely, viridans streptococci) (*2*–*5*). However, 0.6%–5.0% of cases are caused by *Streptococcus pneumoniae* (*4*,*6*–*15*), a common cause of community-acquired pneumonia, otitis, sinusitis, and invasive diseases, especially among persons <2 or >65 years of age and among patients with underlying conditions (*13*,*16*). Invasive pneumococcal disease (IPD) is a major public health problem; reported annual incidence is 7–97 cases/100,000 adult population (*13*).

Key tools in the clinical management of IPD are antimicrobial therapy and vaccination. Because of increased antimicrobial resistance, pneumococcal vaccination is becoming a major public health issue (*17*–*22*). Two types of pneumococcal vaccine are recommended for adults with underlying conditions: a 23-valent pneumococcal polysaccharide vaccine (PPV23) and a 13-valent pneumococcal conjugate vaccine (PCV13). Use of PCVs has reduced the burden of pneumococcal diseases and led to a significant decline in vaccine serotypes in IPD across all age groups. However, the incidence of IPD is still high, which might result primarily from serotype replacement (*21*,*22*). In some countries, age-based guidelines for pneumococcal vaccination have been issued for persons >65 years of age (*16*). During June 2010–2023, public health authorities in France recommended that for adults at risk for IPD (immunocompromised patients, including recipients of solid-organ or hematopoietic stem cell transplants, patients with AIDS, and patients with chronic kidney disease or diabetes mellitus), a dose of PCV13 should be followed by a dose of PPV23 (*23*–*25*) (Table 1). In July 2023, PCV20, which contains 7 more serotypes than PCV13, was authorized in France. Because those serotypes are also in PPV23, PPV23 is no longer recommended (*26*). Of note, a 21-valent pneumococcal conjugate vaccine, which covers serotypes not yet covered by any other vaccine, has also been recently licensed (*27*).

**Table 1:** National health authority guidelines on pneumococcal vaccination for adults at risk for pneumococcal disease, France

Characteristic	2017 guidelines	2023 guidelines
Age ≥65 y	No recommendation	No recommendation
Alcohol use		
Active smoking		
Immunocompromised patients: asplenia or hyposplenia, hereditary immune deficiency, HIV infection, solid organ transplant, hematopoietic stem cell, chronic autoimmune or inflammatory disease treated by immunosuppressive or biological drugs, nephrotic syndrome, or patients treated by chemotherapy for a solid tumor or hematologic malignancy	1 dose of PCV13 + 8 weeks later: 1 dose of PPV23 + 5 years later: 1 dose of PPV23	1 dose of PCV20
Patients with chronic diseases: chronic respiratory disease, severe asthma, heart failure or cyanotic heart disease, renal failure, chronic liver disease, diabetes mellitus, osteomeningeal breach, or cochlear implant		

*PCV13,13-valent pneumococcal conjugate vaccine; PCV20, 20-valent pneumococcal conjugate vaccine; PPV23, 23-valent pneumococcal polysaccharide vaccine.

To determine the clinical and microbiological characteristics of pneumococcal septic arthritis, we retrospectively studied a large series of cases among adult patients during the 2010–2018 conjugate vaccine era in France. In accordance with the legislation on retrospective, observational studies of clinical practice in France, patients’ informed consent was not required. Our study was approved by the French National Data Protection Commission (reference CNIL 2217356v0).

## Patients and Methods

During January 1, 2010–December 31, 2018, we conducted a retrospective study of cases of pneumococcal septic arthritis (PSA) in adults (>18 years of age) reported to 15 university hospital laboratories in France (all members of the Regional Pneumococcal Observatories network). We defined cases of PSA as those in patients with a *S. pneumoniae*–positive culture from joint aspirates or biopsy samples, a pneumococci-positive blood culture with purulent or inflammatory joint aspirates, or both. We used an anonymous form to retrospectively extract patients’ demographic and clinical characteristics (including age, sex, and underlying conditions), microbiological data, medical and surgical treatments, and outcomes (including death) from medical records.

### Statistical Analyses

In a descriptive analysis, we expressed categorical variables as the frequency (percentage) and continuous variables as the mean ± SD or the median (range), depending on data distribution. We analyzed data with the pvalue.io tool (Medistica, https://www.pvalue.io), using χ^2^ and Fisher exact tests. We set the threshold for statistical significance at p<0.05.

## Results

### Population Characteristics

**Figure 1:**
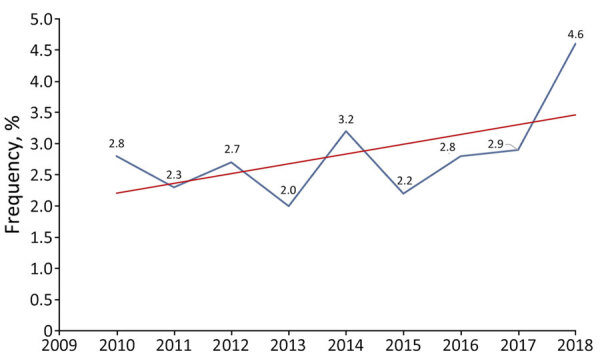
Trends in the frequency of pneumococcal septic arthritis cases among adults as a percentage of all invasive pneumococcal disease cases, by year, France, 2010–2018. Blue line indicates actual values; red line indicates overall trend.

During the 9-year study period, 110 (3.1%) of the 3,501 cases of IPD were ascribed to PSA; the proportion increased slightly over time, albeit not significantly (p = 0.26) (Figure 1). Of the 110 case-patients, 56 were women and 54 men; mean ± SD age was 65.1 ± 14.6 (range 31–93) years. More than half (52.7%, n = 58) of the patients were <65 years of age. On average, women (mean age 67.6) were slightly (but not significantly) older than men (mean age 62.4 years; p = 0.06). The number of cases increased with patient age, and no patients were <30 years of age.

### Clinical Characteristics

Native joint infections (NJIs) accounted for the highest proportion of cases (75%; n = 82), and prosthetic joint infections (PJIs) affected 28 (25%) patients (Table 2). Patients with a PJI were significantly older (mean age 71.6 ± 13 years) than those with an NJI (62.8 ± 14.5 years; p = 0.01). The most common signs/symptoms were pain (88.3%) and edema (45.8%). Fever affected only 26.7% of patients. The median diagnostic delay for PSA was 1 day (range 1–60 days).

**Table 2:** Clinical, treatment-related, and prognostic characteristics of patients with pneumococcal septic arthritis, France, 2010–2018*

Characteristic or finding	Value
Demographic	
Sex	
F	56/110 (50.9)
M	54/110 (49.1)
Age, y, mean ± SD	65.1 ± 14.6
>65 y of age	52/110 (47.3)
Clinical variables	
Pain	30/110 (27.5)
Pain + fever + edema	74/110 (67.9)
Native joint	82/110 (74.6)
Prosthetic joint	28/110 (25.4)
Single joint affected	94/110 (85.5)
Multiple joints affected	16/110 (14.5)
Joints affected	
Knee	50/110 (45.5)
Hip	25/110 (22.7)
Ankle	15/110 (13.6)
Spondylodiscitis	15/110 (13.6)
Wrist	10/110 (9.1)
Shoulder	8 /110 (7.3)
Sacroiliac joint	4/110 (3.6)
Elbow	1/110 (0.9)
Acromioclavicular joint	1/110 (0.9)
Pubic symphysis	1/110 (0.9)
Concomitant infections	
Bacteremia	55/96 (57.3)
Respiratory infection	29/102 (28.4)
Endocarditis	5/102 (4.9)
Meningitis	4/102 (3.9)
Main medical risk factor	
Hematologic malignancy	12/83 (14.4)
Diabetes	11/83 (13.2)
Alcoholism	8/83 (9.6)
Active smoking	8/83 (9.6)
Multiple myeloma	6/83 (7.2)
Solid cancer	6/83 (7.2)
Chronic kidney failure	6/83 (7.2)
Splenectomy	5/83 (6.0)
Heart disease	4/83 (4.8)
Rheumatoid arthritis	3/83 (3.6)
HIV	3/83 (3.6)
Antimicrobial therapy, mean ± SD duration, d	
Duration of intravenous drug therapy	19.1 ± 15.2
Overall duration of antimicrobial therapy	54.1 ± 54.3
Surgery	
Joint drainage or lavage	44/86 (51.2)
Arthrotomy	13/86 (15.5)
Prothesis removal or replacement	16/26 (61.5)
Outcome	
Sequelae	9/82 (11)
Death	8/85 (9.4)

*Values are no. cases/total no. (%) unless otherwise indicated.

Of the 110 patients, 94 had single-joint PSA and 16 had PSA in >2 joints. Multiple-joint PSA was more common in patients with an NJI (93.7%; 15/16 patients) than a PJI (6.2%; 1/16 patients); p<0.01 and was more common among younger patients.

**Figure 2:**
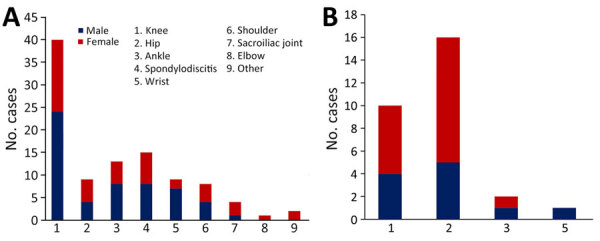
Distribution of pneumococcal joint infections in adults with pneumococcal septic arthritis, by joint and by sex, France, 2010–2018. A) Native joint infections (n = 82); B) prosthetic joint infections (n = 28).

The most commonly involved joint was the knee (45.5%; 50/110 patients), followed by the hip (22.7%; 25/110 patients) (Table 2). The hip was more commonly affected in patients with a PJI (57%, 16/28 patients) than an NJI (11%, 9/82 patients; p<0.001), and the knee was more commonly affected in patients with an NJI (48.8%, 40/82) than a PJI (35.7%, 10/28; p = 0.27), albeit not significantly (Figure 2). Spondylodiscitis was diagnosed for 13.6% (15/110) and sacroiliac joint infection for 3.6% (4/110) of patients; acromioclavicular joint infection was observed in 1 (1%) patient. Among the 16 patients with multiple-joint PSA, infected sites included the wrist (n = 8), ankle (n = 7), shoulder (n = 5), and elbow (n = 1); 9 (56%) of the 16 patients had either ankle or wrist and knee involvement. When considering the PSA site as a function of the patient’s sex, knee involvement was more common among men (53%) than women (39%) but not significantly (p = 0.16). Hip involvement was less common among men (17%) than women (29%) but not significantly (p = 0.28). PSA was more common among women (32%) than men (19%) but not significantly (p = 0.1).

Among the 96 patients for whom blood cultures were performed, bacteremia was found in 55 (57.3%). Bacteremia was significantly more common among patients with an NJI (87%; 48/55) than a PJI (13%; 7/55; p<0.01), more common in knee joints (58%; 32/55) than in hip joints (14.5%; 8/55; p<0.01), and more common among women (58%; 32/55) than men (42%; 23/55; p = 0.036). Bacteremia was more common among patients >65 years of age than among younger patients (Table 3).

**Table 3:** Characteristics of patients with pneumococcal septic arthritis, by age, France, 2010–2018*

Characteristic	No. patients/no. with available data (%)		p value
	Age >65 y, n = 52	Age <65 y, n = 58	
Underlying condition(s)	39/102 (38.2)	44/102 (43.2)	0.2
Multiple-joint infection	3/16 (18.75)	13/16 (81.25)	**0.018**
Prosthesis	18/28 (64.3)	10/28 (35.7)	0.06
Bacteremia	31/55 (56.4)	24/55 (43.6)	**0.03**
Penicillin-nonsusceptible pneumococcus	20/32 (62.5)	12/32 (37.5)	0.06
Serotype			
PCV13 + PPV23	17/34 (50)	17/34 (50)	0.3
Non-PCV13, non-PPV23	8/21 (38)	13/21 (62)	
Death	5/8 (62.5)	3/8 (37.5)	0.27

*Boldface indicates statical significance (p<0.05). PCV13,13-valent pneumococcal conjugate vaccine; PPV23, 23-valent pneumococcal polysaccharide vaccine.

We found that 37.2% (38/102) of patients for whom data were available had prior or concomitant pneumococcal infections; infections mainly affected the respiratory tract (28.4%; 29/102). Of 102 patients, 5 (4.9%) had endocarditis and 4 (3.9%) had meningitis; both conditions were more common among patients with multiple-joint PSA.

### Underlying Conditions and Pneumococcal Vaccination Status

At least 1 risk factor was noted for 83 (81.4%) of the 102 patients for whom data were available, and no risk factors were noted for 19 (18.6%) patients (p<0.0001). The underlying conditions were mainly hematologic malignancies (n = 12), diabetes (n = 11), solid cancers (n = 6), and chronic kidney failure (n = 6). Underlying joint disease was documented for 3 patients, and alcoholism and active smoking were documented for 8 patients. Vaccination status data were available for 32 (29.1%) of the 110 patients, only 7 (21.8%) of whom had been vaccinated against pneumococci (PCV13+PPV23, n = 1; PPV23, n = 6).

### Laboratory Findings

Joint aspirates or biopsy samples were obtained from 109 patients, among whom all samples were inoculated into blood culture vials for 23 patients. Microscopic analysis results were therefore available for only 86 patients. For 81 (94.2%) joint aspirates, the leukocyte count was >10 × 10^9^ cells/mm^3^ (range 10–320 × 10^9^ cells/mm^3^). Gram-staining results were available for 78 (91%) of the 86 joint aspirate samples and revealed gram-positive cocci in 52 (67%).

Of the 109 samples, *S. pneumoniae* was the only isolated pathogen for 107 (98.2%). Pneumococcal bacteremia was detected in 55 (57.3%) of the 96 patients for whom peripheral blood samples had been obtained. *S. pneumoniae* isolates were recovered from joint aspirates or biopsy samples and peripheral blood cultures for 52 (54.2%) of the 96 patients. For 3 patients, a bacteriological diagnosis of PSA was based exclusively on the positive peripheral blood culture.

The Alere BinaxNOW (Abbott Diagnostics, https://www.globalpointofcare.abbott) pneumococcal urinary antigen (PUA) testing was performed for 15% (17/110) of the patients and was positive for 11 (64.7%). Of the 11 patients, 9 (81.2%) had bacteremia.

Antimicrobial susceptibility testing showed that 29.1% (n = 32) of the 110 isolates tested were penicillin-nonsusceptible pneumococcus (PNSP), 7.3% (n = 8) were nonsusceptible to amoxicillin, and 2.7% (n = 3) were nonsusceptible to third-generation cephalosporins. No strain was categorized as being resistant to any of the β-lactams. The PNSP strains were more common, albeit not significantly, among patients >65 years of age (62.5%) than among younger patients 37.5% (p = 0.06) (Table 3) and among patients with a PJI (39.3%) than among patients with an NJI 25.6% (p = 0.17).

**Figure 3:**
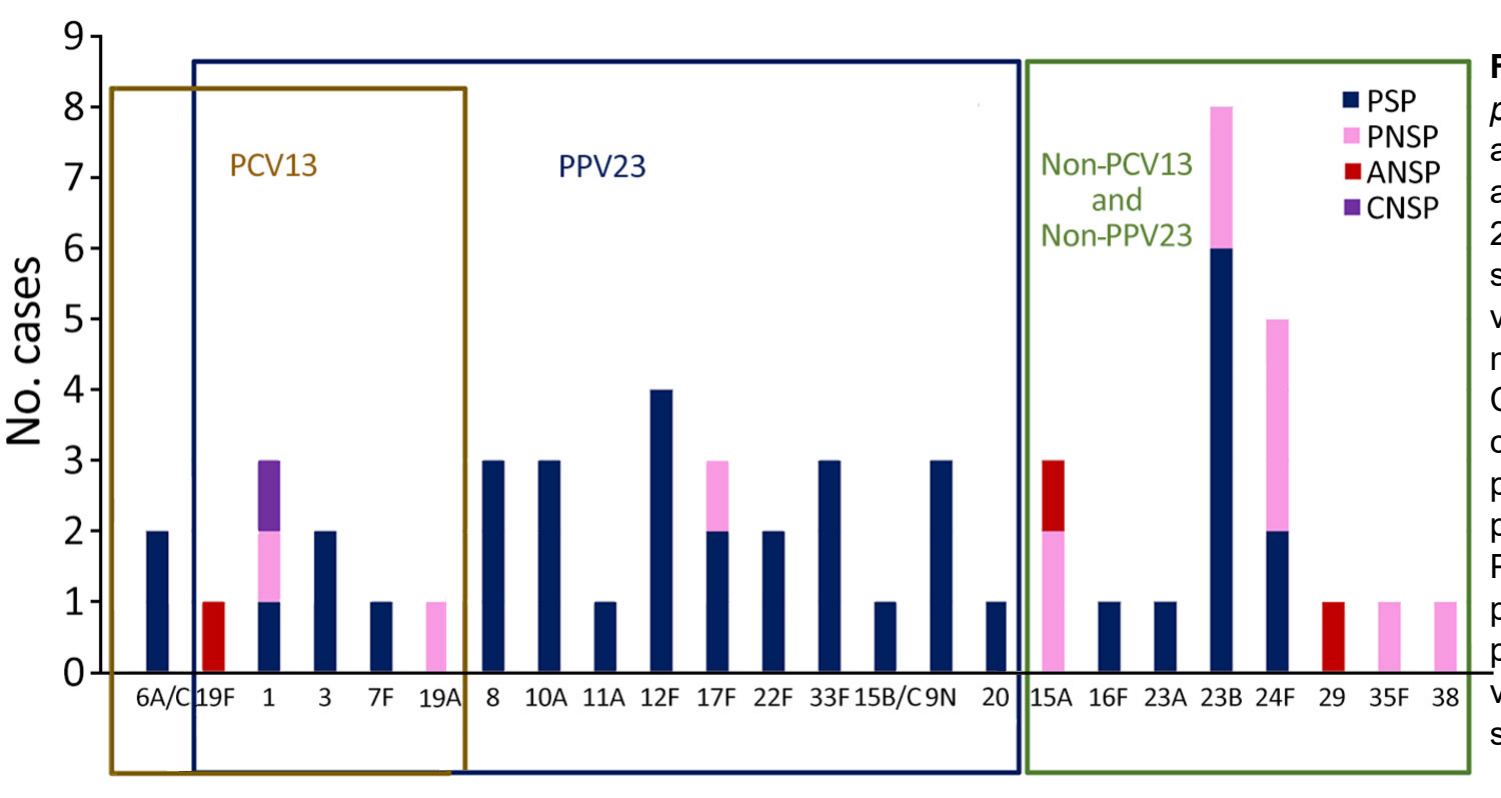
*Streptococcus pneumoniae* serotype distribution and β-lactam susceptibility in adults with septic arthritis, France, 2010–2018. Boxes indicate serotype distribution within different vaccines. ANSP, amoxicillin-nonsusceptible pneumococcus; CNSP, (third-generation) cephalosporin–nonsusceptible pneumococcus; PCV13,13-valent pneumococcal conjugate vaccine; PNSP, penicillin-nonsusceptible pneumococcus; PPV23, 23-valent pneumococcal polysaccharide vaccine; PSP, penicillin (β-lactam)–susceptible pneumococcus.

Serotype data were available for 55 of the 110 strains: 10 (18.2%) were covered by PCV13 (serotypes 1, 3, 6A, 7F, 19A, and 19F), 32 (58.2%) were covered by PPV23 (mainly 8, 9N, 10A, 12F, and 22F), and 21 (38.2%) were not covered by those vaccines (mainly 23B, 24F, and 15A). Vaccination coverage for both PCV13 and PPV23 was 62%. Of the 55 serotyped strains, 16 (29.1%) were PNSP. Of those, 4 (25%) were covered by PCV13 and 5 (31%) were covered by PPV23; 10 (62.5%) were not covered by PCV13 or PPV23 (mainly 15A, 23B, 24F). Three serotypes (15A, 19F, or 29) had low-level resistance to amoxicillin, and 1 isolate (serotype 1) had low-level resistance to third-generation cephalosporins (Figure 3).

### Treatments and Outcomes

Of the 100 patients with available data, 94 (94%) received a combination of 2 intravenous antimicrobial drugs, mainly amoxicillin (65%) or third-generation cephalosporins (24%) and gentamicin (24%) or levofloxacin (22%). Those treatments were followed by oral amoxicillin (75%), levofloxacin (31%), or rifampin (15%), alone or in combination. The mean duration of antimicrobial therapy was longer among patients with a PJI (94.5 days) than patients with an NJI (38.8 days; p<0.01). In addition to antimicrobial therapy, 63 (73%) of the 86 patients with available data underwent surgery. Of the 82 patients with an NJI, 47 (57.3%) underwent >1 surgical intervention (arthrotomy or joint drainage), and the prosthesis was removed or replaced for 16 (61.5%) of the 26 patients with a PJI (Table 2).

Sequelae such as mobility problems and chronic pain were noted for 11% (9/82) of patients with available data, one of whom with an NJI subsequently underwent amputation. The mortality rate was 9.4% (8/85 patients for whom data were available).

## Discussion

Previous studies have reported that PSA accounted for 0.6%–5% of IPD in adults (*4*,*6*–*15*). Consistent with those findings, we found that the overall proportion of PSA to total IPD cases was 3%. That rate increased slightly over the study period; in contrast, the prevalence of IPDs has decreased in France and in most parts of the world because of conjugate vaccine pressure (*21*,*22*,*28*,*29*). The change has contributed to emergence of non-PCV13 serotypes and rebounded incidence of IPD among adults in several countries (*22*,*28*–*31*).

Among adults, IPDs are more frequently encountered in persons >65 years of age, persons with underlying conditions (e.g., hematologic malignancies, diabetes, active smoking, and alcoholism) (*13*,*16*,*17*,*32*,*33*), or both. In our study, 97 (88%) of the 110 patients were >65 years of age, had underlying conditions, or both. Almost half of patients (47.3%) were >65 years of age, a proportion lower than previously reported (62.5%–64%) (*12*,*14*). It has also been suggested that male sex may represent a risk factor for septic arthritis, including PSA (*11*,*12*,*15*,*34*). In our study, however, PSA was equally common among women and men.

In accordance with several previous studies (*6*–*8*,*10*–*12*,*14*,*35*), we found that the most frequently affected joint was the knee. In contrast to other studies in which the shoulder was the second most commonly affected site (7.8%) (*6*,*7*,*9*,*11*,*12*,*15*,*36*,*37*), we found the second most frequently affected joint to be the hip (22.7%). That discrepancy could be because PJIs were excluded in several published studies and because the frequency of PJIs (most of which were hip prosthesis infections) was higher in our study (25%) than that reported in other studies (13%) (*7*–*10*,*12*).

The proportion of patients in our study with spondylodiscitis (13.6%) was also higher than that reported in the literature (0–6.4%) (*6*–*8*,*10*–*12*,*14*,*35*,*38*). The difference might be associated with the possible underdiagnosis of pneumococcal vertebral infections, as suggested by Suzuki et al. (*38*). Although a significant intersex difference was not observed for PSA overall, PJIs were more common among women than men, which has been observed previously (*39*). The intersex difference probably results from the greater life expectancy for women than men in Western countries (*40*). Among adults, multiple joint infections are caused more commonly by *S. pneumoniae* than by other bacterial pathogens (*8*,*9*,*37*). In accordance with data in the literature (*3*,*8*,*11*,*12*,*14*,*32*), we found that multiple-joint PSA affected mostly native joints.

It has been reported that prior or concomitant pneumococcal infections (including meningitis and endocarditis) may be frequent (range 37.5%–67%) among patients with PSA (*6*–*10*,*12*,*14*,*37*). In our study, those infections were noted in 37.2% of patients; meningitis, endocarditis, or both were found in patients with a knee NJI or multiple-joint PSA. The proportion of patients with bacteremia (57.3%) in our study was in accordance with literature values (55%–100%) (*3*,*5*,*6*,*8*–*11*,*14*). However, in contrast to 2 published studies (*9*,*14*) but in agreement with a third (*32*), we observed that bacteremia was more common among patients with an NJI than those with a PJI. The frequency of documented bacteremia emphasizes the value of obtaining blood cultures in addition to joint aspirates or biopsy samples before initiating antimicrobial therapy (*9*,*14*,*37*).

In our study, positive Gram staining (which leads to a rapid diagnosis and narrows the scope of empirical treatment) was noted for 67% and a positive culture was noted for 98% of patients. Our results are consistent with reports in the literature (*5*–*10*,*14*,*37*). In contrast, the sensitivity of the PUA was lower in our study than in the literature (*41*). The discrepancy might result from the fact that PUA testing was not performed for all patients but was perhaps also associated with changes in the distribution of the pneumococcal serotypes (*42*,*43*). Indeed, sensitivity appears to vary among serotypes (e.g., from 33.1% for 23B to 100% for 18C and 20) because of differences in C polysaccharide composition (*42*).

Most of the strains obtained from patients with PSA have been reported as being susceptible to β-lactams (*6*–*10*,*35*). In contrast, we found that 29% of the strains—mostly nonvaccine serotypes such as 23B, 24F, and 15A—had low-level resistance to β-lactams. Our results are consistent with those reported in a single-center study performed in France during the same study period and in agreement with the overall proportion of PNSP among patients with IPD in France during 2010–2021 (27.2%–29.8%) (*14*,*22*). Indeed, in the PCV13 era, the proportion of PNSP is still high in France, and during 2010–2020, the proportion of PNSP among patients with IPD in the United States fell from 21% to 12% (*44*). Those differences can be explained by geographic differences in serotype replacement (*28*,*45*–*47*) and in susceptibility to β-lactams (*28*,*45*).

Given the potential severity of septic arthritis, patients should be hospitalized for early diagnosis and prompt treatment (*2*). In our study, the median time from hospital admission to PSA diagnosis was 1 day, which is consistent with standard of care. However, the interval was often longer for patients with a PJI (median 2 days) or spondylodiscitis (6 days), which might result at least in part from the low specificity of the signs and symptoms. In our study, we found that amoxicillin and third-generation cephalosporins (alone or in combination) are most commonly used to treat PSA (*6*,*7*,*10*,*48*,*49*). To the best of our knowledge, no high-quality, randomized, controlled studies of the optimal treatment duration for septic arthritis have been performed. Thus, the current treatment guidelines are based on expert opinions. Although the optimal treatment duration for PJI is still subject to debate (*32*), 4 weeks of therapy are considered sufficient for uncomplicated PSA (*6*,*8*,*48*). It has also been suggested that 2 weeks of therapy may be adequate for uncomplicated septic arthritis of the small joints, including PSA (*15*). In our study, NJIs always occurred in large joints (except for in 1 patient who had acromioclavicular arthritis), and the mean treatment duration was ≈6 weeks. One can speculate that the course of antimicrobial treatment could be safely shortened for some patients. However, that speculation remains to be confirmed because a lack of data and the small number of patients prevented us from establishing a correlation between occurrence of complications and treatment duration. Antimicrobial therapy may be successful in the absence of drainage. However, the best treatment for septic arthritis is considered to be the combination of drainage and antimicrobial therapy (*1*,*5*–*10*). In our study, arthrotomy and joint drainage were performed for patients with NJIs. For patients with PJIs, the prosthesis (mainly the hip) was removed or replaced, as is often suggested for patients with chronic prosthetic septic arthritis or septic arthritis caused by other bacteria, such as staphylococci (*8*,*32*,*48*,*49*).

The proportion of patients experiencing sequelae in our study (11%) was in accordance with proportions reported elsewhere (11%–40%) (*6*,*10*,*12*). In the literature, the mortality rate for PSA ranges from 19% to 35% (*6*,*8*,*9*), and the risk for death seems to be higher among patients with bacteremia and among patients >60 years of age (*8*). In our study, we found a lower mortality rate (9.4%), and we did not notice a difference in mortality rate as a function of the presence of bacteremia. However, the mortality rate was higher among patients >60 years of age and those with underlying conditions, consistent with previous reports (*8*,*32*). The mortality rate associated with PSA is known to be age-dependent, and the lower mortality rate in our study can be explained by the fact that more than half of our patients were <65 years of age.

In our study, most patients had not received pneumococcal vaccination (despite the presence of underlying conditions), and more than half of the strains isolated were covered by both PCV13 and PPV23. A recent study in France showed that the pneumococcal vaccination rate was very low among adults (4.5%) (*24*), which might result from lack of a defined age threshold for eligible patients, vaccination hesitancy, or both. In our study, about half of the patients were >65 years of age; in several countries, pneumococcal vaccination is recommended for that age group (*16*). Thus, as suggested previously (*24*), an invitation for vaccination at the time of entry into the recommended age group would probably increase the pneumococcal vaccination coverage rate.

Among the limitations of our study are, first, that it was a retrospective study; thus, details of the PSA, immunization status, serotype, antimicrobial therapy, and clinical outcomes were not available for all patients. Second, including data from nonparticipating university hospitals in France, other public-sector hospitals, and private-sector hospitals would probably have yielded a greater number of cases of PSA. Third, our study was based on joint aspirates or biopsy samples that were *S. pneumoniae* culture positive, which probably also led to underestimation of the number of cases of PSA.

In conclusion, although PSA is uncommon in adults, we reported on >100 cases in France, including cases in patients >65 years of age, patients with underlying conditions, and patients with a prosthesis. Some emerging serotypes display a low level of susceptibility to β-lactams and have also emerged among persons with IPDs and community-acquired pneumonia in France and several other countries. Those serotypes are covered by the new generation of PCVs, so vaccination among appropriate age groups should be encouraged. 
